# Update on novel, validly published, and included bacterial taxa derived from nondomestic animals and taxonomic revisions published in 2024

**DOI:** 10.1128/jcm.01070-25

**Published:** 2025-10-17

**Authors:** Claire R. Burbick, Sara D. Lawhon, Trinity Krueger, Elena Ruiz-Reyes, Mallory Lenmark, Ayden Wisney-Leonard, Erik Munson

**Affiliations:** 1 Department of Veterinary Microbiology and Pathology, Washington State University 6760, Pullman, Washington, USA; 2 Department of Veterinary Pathobiology, Texas A&M University 14736, College Station, Texas, USA; 3 Department of Medical Laboratory Science, Marquette University 5505, Milwaukee, Wisconsin, USA; 4 Wisconsin Clinical Laboratory Network Laboratory Technical Advisory Group, Madison, Wisconsin, USA; Medical College of Wisconsin, Milwaukee, Wisconsin, USA

**Keywords:** taxonomy, nomenclature, veterinary microbiology

## Abstract

Description of new and revised taxa originating from non-domestic animal species continues to be pursued. The majority are bacteria isolated from the alimentary system of a variety of healthy wildlife, adding to our understanding of microbial flora present in the normal state. A few new and revised taxa are associated with disease, including a novel *Erysipelothrix* species associated with sepsis in albatross and a revised species, *Allocoenonia anatina* comb. nov., associated with *Riemerella anatipestifera*-like disease in ducks and geese. Representative bacteria from genera that are additionally under study for the remediation of pollutants and bacteria that are related to significant animal or zoonotic pathogens are additionally discussed.

## INTRODUCTION

As researchers continue to describe potentially pathogenic and non-pathogenic bacteria isolated from non-domestic species of animals, a wealth of new microbes is identified. Given the number of non-domestic species and the number of bacteria associated with those species, it is useful to summarize new findings. Although the majority of new bacteria of taxonomic interest are not associated with disease in these animals, they may be of interest as a component of the microbiome (1–13) or have properties that may be of use in scientific or industrial endeavors (1, 14). That being said, newly described bacteria of pathogenic potential, including zoonotic potential, are also included (12, 15, 16). These bacteria may also have implications for domestic spillover and warrant attention.

To facilitate the recognition and adoption of nomenclature changes and as a supplement to past reviews on novel bacterial species discovery/effective publication and nomenclature revisions relative to human clinical sources (17–22), the *Journal of Clinical Microbiology* began in early 2023 to publish papers outlining analogous changes relative to bacterial species derived from non-domestic animals (23–25), domesticated or agricultural veterinary hosts (26–28), and those found in aquatic environments (29, 30). The simple goal of this publication series was to inform and provide data that veterinary microbiology laboratorians can synthesize and integrate into routine laboratory practice. To this end, the *Journal of Clinical Microbiology* has committed to annual publication of new taxon discovery and taxonomic revisions relative to non-domestic and domestic veterinary hosts. Herein, we summarize taxonomic revisions and validly published novel taxa derived from non-domestic hosts formally announced in 2024.

## MATERIALS AND METHODS

Per the International Committee on Systematics of Prokaryotes, validly published novel and revised taxa pertinent to prokaryotic species must satisfy two requirements set forth by the International Code of Nomenclature of Prokaryotes (31). First, original investigations are published in the *International Journal of Systematic and Evolutionary Microbiology* (*IJSEM*). An example relative to the Gram-negative coccus *Neisseria yangbaofengii* is provided (32). In addition, type strains are to be deposited into recognized culture collections in two separate countries. As of January 2018, taxon descriptions in *IJSEM* require genome sequencing data relative to the type strain to be deposited in GenBank (33), with genome accession number included as part of the effective description. The similarity of this sequence is to be assessed against related taxa.

As an alternative to primary publication in *IJSEM*, studies may be published in another journal, with later inclusion in *IJSEM*. One past example relative to bacteria derived from non-domesticated veterinary species is the effective description of the ram-positive bacillus *Corynebacterium marambiense* (34), with subsequent inclusion on an *IJSEM* validation list (35). To be considered for inclusion on a validation list, which is published six times per year, authors must submit a copy of the previously published manuscript to the editorial office of *IJSEM* for confirmation that all elements necessary for valid publication (including culture collection) have been met. Approximately one-half of effectively described, but non-*IJSEM*-published, taxa are not submitted to *IJSEM* for inclusion on validation lists (36).

Non-*IJSEM* journals that have recently published studies providing a valid description of non-domestic animal-derived novel taxa, which may be relevant to the practice of veterinary microbiology, include *Antonie Van Leeuwenhoek*, *Archives of Microbiology*, *Cell Host & Microbe*, *Scientific Reports*, *Systematic and Applied Microbiology*, and *Vector Borne Zoonotic Diseases*. Journals that have recently published studies reflecting revisions in prokaryotic taxonomy relative to non-domestic veterinary clinical material include *Antonie Van Leeuwenhoek* and *Systematic and Applied Microbiology*.

Taxon names validly published through an effective description in *IJSEM* or by inclusion on a validation list may later be considered as synonyms (e.g., by considering them as a heterotypic synonym of a name of the same rank, which has priority, or by proposing a new combination when transferring a species to another genus or when transferring a subspecies to another species). Moreover, Trujillo et al. (37) stated that once a taxon is validly published or included on a validation list, it remains available for use, as long as it is the correct name for the appropriate circumscription, position, and rank. Designations that are transferred to another taxon frequently do not relinquish a validly published status; the majority of prokaryotic nomenclature revisions are due to changes in taxonomic opinion. As an example, the spirochete *Borreliella burgdorferi* currently remains validly (and effectively) published (38, 39) as a synonym of its correct designation *Borrelia burgdorferi* (40). Table headers throughout this document include phrases such as “Previous Designation” and “Revised Designation” to reflect this paradigm.

All issues of *IJSEM* published from January 2024 through December 2024 (including six validation lists) were manually searched for original articles describing novel, validly published species or accepted changes in taxonomic nomenclature. Toward the goal of consistency, only nomenclature with taxonomic status of “correct name” (per data curated by LPSN—List of Prokaryotic names with Standing in Nomenclature; https://lpsn.dsmz.de) was included. On rare occasions, taxa published in *IJSEM* during calendar year 2024 (e.g., *Kaistella faecalis* comb. nov. [41]) were subsequently classified by LPSN as synonym designations (e.g., correct name *Chryseobacterium faecale* [42]) and were thus excluded from further consideration. (Validation lists have also been the source of apparent post-publication reversion to synonym status; *Paramicrobacterium chengjingii* comb. nov [43, 44]; subsequently being deferred to its correct and non-synonym designation of *Microbacterium chengjingii* [45] is presented as one example.) The audit was further filtered by organisms recovered from non-domestic veterinary material. Not included within the definition of non-domestic wildlife animals are companion animals, animals found in agricultural settings, and farmed avian species. Mice derived from wilderness settings, rather than “house mice” or those that are genetically characterized for laboratory settings, are included within this report.

## RESULTS AND DISCUSSION

A compilation of novel taxa recovered from non-domestic veterinary sources stratified by Gram morphology and oxygen growth requirement is presented in Table 1. The phenotypic means of taxa organization within Table 1 appeal to the practicing veterinary microbiologist. Such designations are presented in alphabetic order, rather than chronological order, within each stratification. Table 2 provides alternative taxonomic designations for organisms originally recovered from non-domestic veterinary sources.

**TABLE 1:** Novel, validly published bacterial species recovered from non-domestic wildlife veterinary material reported from January 2024 through December 2024

Scientific Name	Family	Source^*a*^	Growth characteristics	Reference(s)
Gram-positive coccus				
*Streptococcus suis* subsp. *hashimotonensis* subsp. nov.	*Streptococcaceae*	Wound infection of human bitten by boar in Japan	Facultative, non-spore-forming Gram-positive coccus; 1.2- to 1.5 mm diameter α-hemolytic (developing into β-hemolysis over time), convex, circular colonies on sheep blood agar; growth at 30-42ºC; does not tolerate 6.5% NaCl; positive for Lancefield group A antigen, acid phosphatase, D-amygdalin, α-glucosidase, β-glucosidase, β-glucuronidase, α-chymotrypsin, tagatose; negative for α-galactosidase, pyroglutamic acid arylamidase, N-acetyl-β-glucosamidase; susceptibility to bacitracin	(15)^*b*^
Gram-positive bacilli				
*Corynebacterium mendelii* sp. nov.	*Corynebacteriaceae*	Oral cavity of Adélie penguin in Antarctica	Aerobic, non-motile, catalase-positive, oxidase-negative, non-spore-forming Gram-positive bacillus; smooth, creamy, convex, circular colonies with entire margins on tryptone soy agar; growth at 10-37ºC in up to 10% NaCl; positive for β-galactosidase, α-glucosidase, maltose, D-tagatose; negative for DNase, tyrosine hydrolysis, alkaline phosphatase, C4 esterase, leucine arylamidase, glycerol, glycogen; susceptible to ciprofloxacin, gentamicin, vancomycin, clindamycin, tetracycline, linezolid; resistant to penicillin G	(46)
*Erysipelothrix amsterdamensis* sp. nov.	*Erysipelotrichaceae*	16 isolates from heart, liver, lung, and brain of three Indian yellow-nosed albatross chick carcasses on Amsterdam Island (southern Indian Ocean)	Non-motile, catalase-negative, oxidase-negative, non-spore-forming Gram-positive bacillus; small, pinpoint, round, flat, gray, α-hemolytic colonies on 5% horse blood agar; growth at 20-40°C; positive for β-galactosidase, alkaline phosphatase, pyroglutamic acid arylamidase, N-acetyl-β-glucosaminidase, alanyl-phenylalanyl-proline arylamidase; negative for β-glucosidase, arginine dihydrolase, β-glucuronidase, α-galactosidase, mannitol, sorbitol, raffinose, hippurate, urease, maltose, melibiose, sucrose, L-arabinose	(16)
*Nocardioides bizhenqiangii* sp. nov.	*Nocardioidaceae*	Feces of Tibetan antelope (*Pantholops hodgsonii*) in China	Aerobic, non-motile, catalase-positive, oxidase-negative, non-spore-forming Gram-positive short bacillus; 0.5- to 1.3 mm diameter creamy white, circular, convex, smooth colonies on Reasoner’s 2A (R2A) agar; optimal growth at 25 ºC; positive for D-mannitol, D-fructose, starch, acid phosphatase; weakly positive for trypsin, β-glucosidase; negative for D-glucose, gelatin hydrolysis, lactose	(1)
*Stomatohabitans albus* gen. nov., sp. nov.	*Stomatohabitantaceae*	Oral cavity of Steller sea lion (*Eumetopias jubatus*) in China	Facultative, motile, catalase-positive, Gram-positive bacillus; circular, white colonies on brain heart infusion agar; optimal growth at 37 ºC; positive for C4 esterase, C8 esterase, lipase, C14 lipase, leucine arylamidase, valine arylamidase, cystine arylamidase, acid phosphatase, esculin ferric citrate, naphthol-AS-BI-phosphohydrolase, α-ketoglutaric acid	(2)^*c*^
Gram-negative bacilli				
*Pedobacter faecalis* sp. nov.	*Sphingobacteriaceae*	Feces of eland (*Taurotragus oryx*) in the Republic of Korea	Aerobic, non-motile, catalase-positive, oxidase-positive Gram-negative bacillus; white, round colonies on R2A agar; optimal growth at 34°C; no growth on MacConkey agar; positive for cellulose, starch hydrolysis, D-mannose, D-glucose, maltose, valine arylamidase; negative for L-arabinose, β-glucuronidase, β-glucosidase, α-glucosidase, α-fucosidase	(3)
*Rheinheimera faecalis* sp. nov.	*Chromatiaceae*	Feces from white rhinoceros (*Ceratotherium simum*) in South Korea	Aerobic, motile, oxidase-positive, catalase-positive Gram-negative bacillus; colonies propagated on R2A, trypticase soy, marine, nutrient agars; no growth on MacConkey agar; optimal growth at 30 ºC; positive for nitrate reduction, cellulose, N-acetyl-β-glucosaminidase, gelatin hydrolysis, D-maltose, alkaline phosphatase; weakly positive for leucine arylamidase, trypsin; negative for D-glucose, C4 esterase, C8 esterase, lipase, acid phosphatase	(4)^*b*^
*Rhinopithecimicrobium faecis* gen. nov., sp. nov.	*Sphingobacteriaceae*	Feces from black-and-white snub-nosed monkey (*Rhinopithecius bieti*) in China	Aerobic, non-motile, catalase-positive, oxidase-positive, Gram-negative oval-shaped bacterium; yellow colonies propagated on Columbia agar; optimal growth at 20-30ºC; positive for L-rhamnose, glycerol, C4 esterase, cystine arylamidase, α-chymotrypsin, β-galactosidase; negative for sucrose, C14 lipase, L-arabinose, D-xylose; reported susceptible to ampicillin, cefoperazone, ciprofloxacin, clindamycin, gentamycin; resistant to nalidixic acid, aztreonam, vancomycin	(5)^*b*^
*Sphingobacterium zhuxiongii* sp. nov.	*Sphingobacteriaceae*	Isolates (2) from feces of Tibetan gazelle (*Procapra picticaudata*) in China	Aerobic, non-motile, oxidase-negative, catalase-positive Gram-negative bacillus; yellow, mucoid, convex, round, smooth colonies on tryptic soy agar; optimal growth at 30°C; positive for *N*-acetyl-β-glucosaminidase, D-adonitol, leucine arylamidase, β-glucosidase; weakly positive for C4 esterase, C8 esterase lipase, D-lyxose, tagatose; negative for D-mannitol, C14 lipase, α-chymotrypsin	(6)
*Stenotrophomonas forensis* sp. nov.	*Lysobacteraceae*	Cloacal swab	Novel taxonomic designation within *Stenotrophomonas maltophilia* complex reflects an Isleria hauxwelli isolate previously classified as *S. maltophilia*; aerobic, non-motile Gram-negative bacillus; 1.5- to 2.0 mm smooth, non-hemolytic colonies on trypticase soy agar with 5% sheep blood agar; optimal growth at 35°C; non-lactose-fermentative colonies on MacConkey agar; positive for β- *N*-acetyl-glucosaminidase, β-glucosidase, lipase, sodium citrate, L-lactate alkalinization, succinate alkalinization; negative for maltose, malonate	(7)
Gram-positive anaerobes				
*Adlercreutzia aquisgranensis* sp. nov.	*Eggerthellaceae*	Gutcontent of wild mouse	Limited phenotypic characterization provided; Gram reaction and atmospheric requirement predicted by genus assignment	(8)^*d*^
*Adlercreutzia shanghongiae* sp. nov.	*Eggerthellaceae*	Isolates (2) from intestinal contents of plateau pika (*Ochotona curzoniae*) in China	Anaerobic, non-motile, catalase-negative, oxidase-negative, non-spore-forming Gram-positive bacillus; 1.0- to 1.2 mm diameter pale white, moist, translucent, convex colonies with entire edges on modified brain heart infusion agar with blood; optimal growth at 37 ºC; does not tolerate 2% bile; negative for L-arginine arylamidase, proline arylamidase, phenylalanine arylamidase, leucine arylamidase, tyrosine arylamidase, alanine arylamidase, glycine arylamidase, histidine arylamidase, serine arylamidase	(9)
*Adlercreutzia wanghongyangiae* sp. nov.	*Eggerthellaceae*	Isolates (2) from intestinal contents of plateau pika (*Ochotona curzoniae*) in China	Anaerobic, non-motile, catalase-negative, oxidase-negative, non-spore-forming Gram-positive bacillus; 1.0 mm diameter pale white, moist, semi-translucent, convex colonies with entire edges on modified brain heart infusion agar with blood; optimal growth at 37 ºC; tolerates 2% bile; positive for L-arginine arylamidase, proline arylamidase, phenylalanine arylamidase, leucine arylamidase, tyrosine arylamidase, alanine arylamidase, glycine arylamidase, histidine arylamidase, serine arylamidase	(9)
*Clostridium lamae* sp. nov.	*Clostridiaceae*	Alpaca feces in China	Anaerobic, non-motile, catalase-negative, oxidase-positive, non-spore-forming Gram-negative bacillus; 1- to 3 mm diameter translucent, milky white, circular, convex, entire colonies on yeast extract-casein hydrolysate-fatty acid agar; optimal growth at 37 ºC; positive for D-mannitol, D-xylose, L-arabinose, D-glucose, esculin, glycerol, salicin; negative for β-glucosidase, indole, urease	(10)^*e*^
Gram-negative anaerobes				
*Bacteroides rhinocerotis* sp. nov.	*Bacteroidaceae*	Feces from rhinoceros in China	Anaerobic, non-motile, catalase-negative, oxidase-negative, non-spore-forming Gram-negative bacillus; 1- to 3 mm diameter whitish, translucent, circular, convex colonies on modified Gifu anaerobic agar; optimal growth at 37 ºC; positive for chymotrypsin, acid phosphatase, α-fucosidase,indole, D-mannitol, D-sorbitol, D-trehalose; negative for β-glucuronidase, α-fucosidase, glycerol, L-rhamnose, leucine arylamidase, tyrosine arylamidase, arbutin, *N*-acetyl-D-glucosamine	(11)^*d*^
*Odoribacter lunatus* sp. nov.	*Odoribacteraceae*	Cecal slurry of wild mouse	Limited phenotypic characterization provided; Gram reaction and atmospheric requirement predicted by genus assignment	(8)^*d*^
Curved bacteria				
*Campylobacter californiensis* sp. nov.	*Campylobacteraceae*	Isolates (4) from feral swine feces in the United States	Studied in association with *Campylobacter* spp. outbreak in raw/milk colostrum from a California dairy; microaerophilic, motile, oxidase-positive, catalase-negative, slightly-curved Gram-negative bacillus and coccoid cells; 1- to 2 mm diameter glistening, opaque, convex, circular, entire colonies on anaerobe basal agar with blood; growth at 37–42ºC in microaerophilic conditions and at 30 ºC in anaerobic conditions: positive for nitrate, selenite, glycine; negative for alkaline phosphatase, hippurate, indoxyl acetate, urease; H_2_S on triple sugar iron agar is variable; resistant to nalidixic acid and cephalothin	(12)
*Campylobacter porcelli* sp. nov.	*Campylobacteraceae*	Wild boar feces in United States	Microaerophilic, motile, oxidase-positive, catalase-positive Gram-negative curved bacillus; 1- to 2 mm diameter opaque, glistening, circular, convex, entire colonies on anaerobe basal agar with blood after 3 days; growth at37°C and 42°C (no growth at 30°C; no growth aerobically); positive for nitrate reduction, selenite reduction, alkaline phosphatase; negative for urease, hippurate hydrolysis, indoxyl acetate; resistant to nalidixic acid, cephalothin	(13)
*Campylobacter vicugnae* sp. nov.	*Campylobacteraceae*	Isolates (4) from sheep feces in Scotland, China	Microaerophilic, motile, oxidase-positive, catalase-positive Gram-negative spiral/curved bacillus; 1- to 2 mm diameter opaque, glistening, circular, convex, entire colonies on anaerobe basal agar with blood after 3 days; growth at 37°C and 42°C (no growth at 30°C; no growth aerobically); positive for nitrate reduction, alkaline phosphatase; negative for urease, hippurate hydrolysis, indoxyl acetate, selenite reduction; resistant to nalidixic acid; variable resistance to cephalothin	(13)
Mollicute				
*Mycoplasma bradburyae* sp. nov.	*Mycoplasmataceae*	Isolates from Larus michahellis (4), *Chroicocephalus ridibundus* (1), *Calonectris borealis* (1) seabirds in Italy, South Africa, Spain	Cell wall-deficient, motile, pleomorphic (exhibit flask shape or tip-like structure) bacterium; passage through 450- and 220 nm membranes; cultivated in modified Hayflick medium; optimal growth at 37-42.5°C; serum or sterol required for growth; capable of hemolyzing sheep erythrocytes; produces acid from glucose and mannose; negative for urease and arginine dihydrolase	(47)^*d*^

^*a*^ Number of isolates from a clinical source is 1, except where shown in parentheses.

^*b*^ Validly published by inclusion in Validation List no. 220 (48).

^*c*^ Validly published by inclusion in Validation List no. 219 (49).

^*d*^ Validly published by inclusion in Validation List no. 216 (50).

^*e*^ Validly published by inclusion in Validation List no. 218 (51).

**TABLE 2:** Revised bacterial taxon relative to non-domestic wildlife animal sources of veterinary material from January 2024 through December 2024

Previous designation	Revised designation	Other information	Reference
Gram-positive cocci			
*Jeotgalicoccus pinnipedialis*	*Phocicoccus pinnipedialis* comb. nov.	Initial description of the *J. pinnipedialis* taxon from the southern elephant seal (*Mirounga leonina*) found in (52)	(53)^*a*^
*Kocuria rosea*	*Kocuria rosea* subsp. *rosea* comb. nov.	Subset of *K. rosea* strains revised to *Kocuria rosea* subsp. *rosea* comb. nov.; *K. rosea* remains a valid and correct taxon; examples of *K. rosea* incidence in bullfrogs found in (54)	(55)
*Kocuria uropygialis*	*Rothia uropygialis* comb. nov.	Initial description of the *K. uropygialis* taxon from preen glands of great spotted woodpeckers (*Dendrocopos major*) found in (56) and accepted (57)	(55)
*Kocuria uropygioeca*	*Rothia uropygioeca* comb. nov.	Initial description of the *K. uropygioeca* taxon from preen glands of great spotted woodpeckers (*Dendrocopos major*) found in (56) and accepted (57)	(55)
Gram-negative bacillus			
*Coenonia anatina*	*Allocoenonia anatina* comb. nov.	Initial description of the *C. anantina* taxon from ducks and geese with respiratory illness in Germany found in (58)	(59)
Gram-positive anaerobe			
*Clostridioides mangenotii*	*Metaclostridioides mangenotii* comb. nov.	Taxonomic revision to *Clostridioides mangenottii* described in (60) and accepted (61); initial publication of the *Clostridium mangenotii* taxon found in (62); recovery from timber rattlesnake found in (63)	(64)
Obligate intracellular bacterium			
*Chlamydophila caviae*	*Chlamydia caviae* comb. nov	Initial valid publication of the *Chlamydophila caviae* taxon found in (65); pathogenic incidence in crocodiles documented in (66)	(67)^*b*^

^*a*^ Validly published by inclusion in Validation List no. 215 (68).

^*b*^ Validly published by inclusion in Validation List no. 218 (51).

### Novel taxa

The majority of new taxa originate from the alimentary tracts of multiple wild animal species with no overt disease. These bacteria are primarily Gram-positive and -negative bacilli and include both facultative anaerobes and strict anaerobes (Table 1). Family *Sphingobacteriaceae* was overrepresented, including the Gram-negative bacilli *Pedobacter faecalis* sp. nov., *Rhinopithecimicrobium faecis* gen. nov., sp. nov., and *Sphingobacterium zhuxiongii* sp. nov., identified from feces of an eland (*Taurotragus oryx*), a black-and-white snub-nosed monkey (*Rhinopithecius bieti*), and Tibetan gazelle (*Procapra picticaudata*), respectively (3, 5, 6). The ram-positive anaerobe family *Eggerthellaceae* has multiple novel taxonomic descriptions of intestinal origin, including *Adlercreutzia aquisgranens* sp. nov., identified from a wild mouse (8), and both *Adlercreutzia shanghongiae* sp. nov. and *Adlercreutzia wanghongyangiae* sp. nov., identified from plateau pika (*Ochotona curzoniae*) (9). *Adlercreutzia* spp. are notable not only for the requirement of an anaerobic environment but also for the toleration of 2% bile and the lack of spore formation.

Although new taxa may be of interest for their role as a component of the microbial flora of wildlife, two are found within genera that are under assessment for use in the mitigation of pollutants. *Nocardioides bizhenqiangii* sp. nov., identified from the feces of Tibetan antelope (*Pantholops hodgsonii*) (1), is a member of a genus with numerous species under study for use in the bioremediation of petroleum and other carbon- and nitrogen-based compounds (14). *Rheinheimera faecalis* sp. nov. was identified from the feces of a white rhinoceros (*Ceratotherium simum*) (4), whereas a related but not validly published species, “*Rheinheimera metallidurans,*” is under investigation for its ability to degrade mercury (69). As more bacteria are described from animal and environmental sources, the possibility of harnessing the metabolic capacity of these novel organisms could aid in restoring the health of the environments from which they were found.

Multiple new taxa derived from non-domestic hosts are either associated with disease or are members of a genus associated with disease in non-domestic or domestic species. *Erysipelothrix amsterdamensis* sp. nov., a ram-positive bacillus, was identified from albatross (*Thalassarche carteri*) chicks with lesions compatible with bacterial sepsis (16). Concern for additional infectious agents impacting albatross populations on Amsterdam Island (70, 71) beyond *Pasteurella multocida*, a well-described pathogen of wild and domestic birds (72), resulted in surveillance of deceased chicks. Identification of an *Erysipelothrix* species was highly suggestive, as *Erysipelothrix rhusiopathiae* is a well-known pathogen of avian species (73). In fact, the isolates obtained from the albatross chicks were originally identified as *E. rhusiopathiae* based on colony morphology and MALDI-TOF mass spectrometry with high confidence. Genomic analysis determined the average nucleotide identity (ANI; Calculator for OrthoANIu) between the isolates obtained from the chicks and *E. rhusiopathiae* (determined to be the most closely related neighbor) to be 95%. Interestingly, the representative strain could be identified as *E. rhusiopathiae* using organism-specific PCR (74) that targeted a non-coding region downstream of the 5S rRNA coding region. As commonly used identification methods misidentified *E. amsterdamensis* sp. nov., care must be taken in the evaluation of bacteria that yield an identification of *E. rhusiopathiae* from wild bird sources, which could have epidemiologic significance. The authors indicate that *E. amsterdamensis* sp. nov. can be differentiated from near neighbors by using molecular and phylogenetic methodology.

A second ram-positive bacillus, *Corynebacterium mendelii* sp. nov., is of interest as other *Corynebacterium* spp. have been associated with disease in the animal species of origin (46). Multiple reports have described diphtheric stomatitis, typified by fibrinopurulent exudative lesions of the oral cavity, weight loss, and (in extreme cases) death associated with *Corynebacterium megadyptis* as well as another novel *Corynebacterium* spp. in yellow-eyed penguins (*Megadyptes antipodes*) (75, 76). Although *C. mendelii* sp. nov. was isolated from the oral cavity of a healthy Adelie penguin (*Pygoscelis adeliae*) (46), it adds to the understanding of oral flora in healthy penguins and may aid in interpreting the significance of *Corynebacterium* spp. with more pathogenic significance. For example, *C. mendelii* sp. nov. lacks genes encoding a pilus used in attachment and associated with virulence in corynebacteria but did have some homologous genes that have been associated with *Corynebacterium* spp. found in penguins with diphtheritic stomatitis. In contrast to *E. amsterdamensis* sp. nov., *C. mendelii* sp. nov. can be differentiated from its nearest neighbors (including *Corynebacterium rouxii* and *Corynebacterium epidermidicanis*) using phenotypic characteristics, including growth at 6.5% and 10% NaCl, as well as utilization of maltose, D-tagatose, and β-galactosidase.

Within the family *Mycoplasmataceae*, a new taxon has been described, *Mycoplasma bradburyae* sp. nov. isolated from a number of different species of seabirds in Europe and South Africa (47). Interest in surveying wild birds for mycoplasmas is largely due to the lack of knowledge of what constitutes normal flora in wild birds and the pathogenic and opportunistic nature of some mycoplasmas, like *Mycoplasma mycoides* subsp. *mycoides*, *Mycoplasmopsis* (*Mycoplasma*) *synoviae,* and *Mycoplasmopsis* (*Mycoplasma*) *bovis*. Mycoplasmas are unique bacteria as they lack a cell wall and can be difficult to culture, requiring specialized media tailored to individual species. In this case, *M. bradburyae* sp. nov. was cultured in modified Hayflick, SP4-II, and commercially produced avian mycoplasma media—indicating that it is amenable to growth in common mycoplasma medium formulations as well as in aerobic and microaerophilic conditions. Genetic characterization of the novel isolate demonstrated that it was phylogenetically distinct. Closest ANI clustering was with *Mycoplasmoides* (*Mycoplasma*) *gallisepticum* (76.10%), *Mycoplasma tullyi* (76.10%), *Mycoplasma imitans* (76.85%), and a *M. gallisepticum*-like organism (75.90%). It is of note that *M. bradburyae* sp. nov. is closely related to *M. gallisepticum*, a significant pathogen of poultry. *M. gallisepticum* causes significant morbidity and mortality, generally due to respiratory infection in commercial chicken and turkey production systems. However, it also causes significant disease in other commercial bird production (e.g., pheasants) and has had devastating impacts on some species of wild birds (e.g., house finches) (77–79). Although *M. bradburyae* sp. nov. appears not to be associated with clinical disease, data indicated that genes related to adhesion and motility were present. However, these determinants lacked homology when compared to *M. gallisepticum* MGC2 (80) and *vlhA* (81), which are associated with virulence in birds.

Finally, two important non-domestic host-derived taxa are mentioned only briefly as they are reviewed in detail in the sister domestic animal species taxonomic update (82). The first, *Streptococcus suis* subsp. *hashimotonensis* subsp. nov., isolated from a human wound sustained after the bite of a wild boar, is of interest as *Streptococcus suis* is an important pathogen of domestic swine and is zoonotic (15). The second is related to a validation list entry during 2024 (48, 83) that has since reverted to synonym status and is not included in Table 1. *Borrelia nietonii* sp. nov., previously classified as *Borrelia hermsii*, is the causative agent of relapsing fever borreliosis. *B. nietonii* sp. nov. was isolated from wild rodents and ticks in the western United States and Canada.

### Taxonomic revisions

Few revisions were published, with the most notable impacting genus *Kocuria*, a Gram-positive coccus. *Kocuria uropygialis* and *Kocuria uropygioeca*, isolated from the preen glands of great spotted woodpeckers (*Dendrocopos major*), were revised to *Rothia uropygialis* comb. nov. and *Rothia uropygioeca* comb. nov. (55). This change highlights the difficulty in differentiation of genera within the family *Micrococcaceae*, given that there is overlap in basic characteristics of cell morphology and metabolic needs, as well as ubiquity of habitat. Given these difficulties, 16S rRNA gene and average amino acid identity analysis clustered the two previously designated *Kocuria* species with the *Rothia* genus, supporting the revision.

A ram-negative bacillus of clinical significance, *Coenonia anatina*, was revised to *Allocoenonia anatina* comb. nov. after it was determined that the *C. anatina* designation was a later homonym of a validly published name, thus violating Principle two and Rule 51b(4), which ensures that newly proposed names are not later homonyms of names in use in bacteriology, botany, or zoology (59). *C. anatina* was originally identified in *Riemerella anatipestifera*-like outbreaks in ducks and geese (58). Both *Riemerella* and *Allocoenonia* genera fall within the order *Flavobacteriales* but are in different families, *Weeksellaceae* and *Flavobacteriaceae*, respectively. *R. anatipestifera* is an important pathogen of wild and farmed ducks and geese, causing significant clinical disease, including its most severe manifestation of fibrinous serositis (84). Interestingly, there are no additional published reports of *A. anatina,* and the organism appears to be easily differentiated from *R. anatipestifera* via biochemical and genetic modalities.

## CONCLUSION

Our understanding of bacterial populations associated with non-domestic animals continues to be pursued regardless of any association with disease. Much of what is studied is of alimentary origin, where there is an obvious wealth of information, which can further our understanding of health in those animals, or perhaps be of use for human pursuits. While few pathogens were described, these findings are important as we manage wild populations under pressure from other infectious agents, climate change, and human encroachment. The authors will continue to review and summarize new and revised taxa from non-domestic animals to ensure these findings are more widely appreciated.
